# Evaluation of cardiovascular risk factors in long-term survivors of adult- and childhood-onset brain tumours: a pilot study

**DOI:** 10.1530/EC-22-0491

**Published:** 2023-07-12

**Authors:** Nikolaos Kyriakakis, Marilena Giannoudi, Satish S Kumar, Khyatisha Seejore, Georgios K Dimitriadis, Harpal Randeva, Adam Glaser, Michelle Kwok-Williams, Georgina Gerrard, Carmel Loughrey, Ahmed Al-Qaissi, Ramzi Ajjan, Julie Lynch, Robert D Murray

**Affiliations:** 1 Department of Endocrinology, Leeds Centre for Diabetes & Endocrinology, Leeds Teaching Hospitals NHS Trust, Leeds, UK; 2 Leeds Institute for Cardiovascular and Metabolic Medicine (LICAMM), University of Leeds, Leeds, UK; 3 Department of Endocrinology, King’s College Hospital NHS Foundation Trust, Denmark Hill, London, UK; 4 Warwickshire Institute for the Study of Diabetes, Endocrinology and Metabolism (WISDEM), University Hospitals Coventry and Warwickshire NHS Trust, Coventry, UK; 5 Pediatric Oncology, Leeds General Infirmary, Leeds Teaching Hospitals NHS Trust, Leeds, UK; 6 Leeds Institute of Medical Research, University of Leeds, UK; 7 Clinical Oncology, Leeds Cancer Centre, Leeds Teaching Hospitals NHS Trust, Leeds, UK

**Keywords:** brain tumour, vascular risk, body composition, lipids, insulin resistance

## Abstract

**Background:**

Survivors of childhood brain tumours (SCBT) and teenage and young adult cancer survivors have an adverse cardiovascular risk profile, which translates into an increased vascular mortality. Data on cardiovascular risk profiles in SCBT are limited, and furthermore, there are no data in adult-onset (AO) brain tumours.

**Patients and:**

*methods*: Fasting lipids, glucose, insulin, 24-h blood pressure (BP), and body composition were measured in 36 brain tumour survivors (20 AO; 16 childhood-onset (CO)) and 36 age- and gender-matched controls.

**Results:**

Compared with controls, patients had elevated total cholesterol (5.3 ± 1.1 vs 4.6 ± 1.0 mmol/L, *P* = 0.007), LDL-C (3.1 ± 0.8 vs 2.7 ± 0.9 mmol/L, *P* = 0.011), insulin (13.4 ± 13.1 vs 7.6 ± 3.3 miu/L, *P* = 0.014), and increased insulin resistance (homeostatic model assessment for insulin resistance (HOMA-IR) 2.90 ± 2.84 vs 1.66 ± 0.73, *P* = 0.016). Patients showed adverse body composition, with increased total body fat mass (FM) (24.0 ± 12.2 vs 15.7 ± 6.6 kg, *P* < 0.001) and truncal FM (13.0 ± 6.7 vs 8.2 ± 3.7 kg, *P* < 0.001).

After stratification by timing of onset, CO survivors showed significantly increased LDL-C, insulin, and HOMA-IR compared with controls. Body composition was characterized by the increased total body and truncal FM. Truncal fat mass was increased by 84.1% compared with controls. AO survivors showed similar adverse cardiovascular risk profiles, with increased total cholesterol and HOMA-IR. Truncal FM was increased by 41.0% compared with matched controls (*P* = 0.029). No difference in mean 24-h BP was noted between patients and controls irrespective of the timing of cancer diagnosis.

**Conclusion:**

The phenotype of both CO and AO brain tumour survivors is characterized by an adverse metabolic profile and body composition, putatively placing long-term survivors at increased risk of vascular morbidity and mortality.

## Introduction

Malignant brain tumours affect both children and adults, although have markedly different long-term outcomes. Five-year survival rates of 73% have been reported following multi-modality treatment in children diagnosed with malignant brain tumours before the age of 15 years (1). Not dissimilar outcomes are observed in teenage and young adults (15–40 years), in whom 5-year survival approaches 68%. In contrast, considerably lower 5-year survival rates of up to 20% are observed in adults diagnosed with malignant brain tumours after the age of 40 years (1). With advances in cancer therapies, however, survival rates in adult brain tumour patients have continuously improved over the last four decades (2).

Increasing survival from childhood cancer has led to the recognition that exposure to multimodal cancer therapy is associated with an increased risk of long-term morbidity and late mortality. Late-onset adverse sequelae in childhood brain tumour survivors (SCBT) included increased mortality, subsequent cancers, endocrinopathies, chronic medical conditions, neurocognitive impairment, and worse socioeconomic outcomes (3, 4). The excess mortality of SCBT predominantly relates to the recurrence or progression of the primary disease; however, an increase in cardiovascular mortality (3) and risk of early and late-occurring stroke (4, 5) are also observed. There are limited cross-sectional studies evaluating conventional vascular risk factors in SCBT (6, 7); however, the data reported in these studies support a mechanism for the increased late-occurring vascular mortality. Far fewer data exist for adult brain tumour survivors, likely reflecting the guarded prognosis of these individuals. Data in teenage and young adult (15–39 years) brain tumour survivors show increased vascular morbidity (8, 9), however, there are no data in adult survivors diagnosed with malignant brain tumours at an older age. Furthermore, there are no data in either teenage and young adults, or older adults, regarding vascular risk factors.

In the current pilot study, we characterized cardiovascular risk factors in long-term brain tumour survivors of both adult-onset (AO) and childhood-onset (CO), with a secondary aim of determining if a significant dichotomy exists in the prevalence of these risk factors between brain tumour survivors of AO and CO.

## Methods

### Participants’ recruitment

We conducted a cross-sectional pilot study in long-term survivors of primary brain tumours. Individuals with a history of malignant brain tumours of both CO (age ≤ 18 years) and AO (age > 18 years) were invited to participate in the study. Patients were recruited from the adult and childhood late-effects oncology clinics at Leeds Teaching Hospitals NHS Trust. The study was approved by the local regional ethics committee (York Research Ethics Committee, 07/Q1108/45) and conducted in line with the ethical requirements set out in the Declaration of Helsinki and the ICH Harmonised Guideline for Good Clinical Practice. All subjects gave their written, informed consent to participate.

Inclusion criteria were (i) diagnosis of a malignant brain tumour during childhood or adult life; (ii) age 16–60 years at enrolment; (iii) treatment with cranial radiotherapy at least 2 years prior to enrolment; and (iv) capacity to provide written informed consent. Exclusion criteria were (i) active malignancy in the last 2 years; (ii) treatment with lipid-lowering agents; (iii) treatment with anti-platelet medication or anticoagulants; (iv) oestrogen-containing contraceptives; and (v) significant renal (creatinine > 120 umol/L) or hepatic impairment (alanine transaminase greater than twice upper limit of normal). A healthy control group was recruited from an electronic advertisement to the Leeds Teaching Hospitals Staff and Medical and Health Care students who attend the University of Leeds. Relatives of patients were invited to participate through an advertisement displayed at the outpatient department. The control group were individually matched to the patients for gender and age (±2 years).

Details of patients’ past medical history were collected using the Patient Pathway Manager (the electronic patient records for Leeds Teaching Hospitals NHS Trust). Results of all endocrine investigations between the time of radiotherapy and the time of participation in the study were retrieved using the Pathology Results Server.

### Anthropometric assessment

Participants attended the endocrine unit following an overnight fast. Height was measured using a wall-mounted stadiometer. Measurements at the waist (mid-point between the iliac crest and lowest rib) and hip (level of the greater trochanter) were undertaken. Weight and body composition were analysed by bioimpedance using a Tanita TBF 300MA monitor (Tanita, Manchester, UK). Regional fat mass was estimated using Harpenden skin callipers at the biceps, triceps, infrascapular, and suprailiac regions. A 12-lead electrocardiogram was undertaken after 15 min of recumbent rest. A 24-h blood pressure (BP) monitor (Welch Allyn, London, UK) was fitted to the patient’s arm at the end of the study visit.

### Metabolic and endocrine investigations

A morning blood sample following an at least 8-h overnight fast was taken for measurement of serum lipid profile (total cholesterol, HDL-cholesterol, LDL-cholesterol, and triglycerides) and glucose metabolism (serum insulin and glucose). Insulin sensitivity was calculated using the homeostatic model assessment for insulin resistance (HOMA) equation (fasting plasma glucose (mmol/L) × fasting serum insulin (mU/L) divided by 22.5). The prevalence of metabolic syndrome was determined according to the International Diabetes Federation criteria (10).

Basal serum values of the anterior pituitary hormones in morning blood samples were used to establish luteinizing hormone (LH)/follicle-stimulating hormone (FSH), thyroid-stimulating hormone (TSH), and prolactin status in all patients. Additionally, the insulin tolerance test (ITT) or the glucagon stimulation test, where the ITT was contraindicated, was used to assess the integrity of the growth hormone (GH) and hypothalamic–pituitary–adrenal axes. Insulin-like growth factor 1 (IGF1) and thyroid function tests were also performed in the control group.

### Laboratory assays

Total cholesterol, HDL cholesterol, and triglycerides were measured by the ADVIA Chemistry Cholesterol Concentrated assay, ADVIA Chemistry Direct HDL Cholesterol, and ADVIA Chemistry Triglycerides_2 Concentrated assay, respectively, while LDL-cholesterol levels were calculated using the Friedewald equation. LDL was not determined if serum triglycerides were greater than 4.0 mmol/L. Serum glucose was measured by an enzymatic assay based on the method by Slein, using hexokinase and glucose-6-phosphate dehydrogenase enzymes. Serum insulin levels were determined using a one-step chemiluminescent immunoassay (ARCHITECT Insulin assay; Abbott Laboratories). The intra-assay variation and inter-assay variations were less than 2.7%. The ARCHITECT insulin assay has a sensitivity of ≤1.0 μU/mL.

GH, IGF1, and SHBG were measured using Siemens Immulite 2000 (GH calibrated against WHO NIBSC IS 98/574). Cortisol, prolactin, and oestradiol were measured by radioimmunoassay on the Siemens Advia Centaur. LH, FSH, TSH, and free thyroxine were measured by chemiluminescence using Advia Centaur, and testosterone was measured using isotope-dilution liquid chromatography-tandem mass spectrometry. All assays were performed in the routine clinical biochemistry laboratories within the Leeds Teaching Hospitals NHS Trust and have been regularly validated by internal quality control and external quality assessment.

### Statistics

The primary end-point was body composition, specifically percentage fat mass. Secondary end-points included additional measures of body composition and cardiovascular risk factors (serum lipids, insulin sensitivity, and BP). Statistical analysis was performed using SigmaPlot version 12.5. The difference between the two groups was analysed using unpaired *t*-tests or Mann–Whitney rank sum test for parametric and non-parametric data, respectively. Univariate analysis was performed using Pearson’s and Spearman’s analysis for parametric and non-parametric data, respectively. Multivariate linear regression was used to determine factors impacting upon cardiovascular risk factors within the cohort. A *P*-value <0.05 was considered as significant.

## Results

A total of 36 patients (15 female, mean age 30.9 ± 13.9 years) and 36 controls (15 female, mean age 31.5 ± 13.4 years) were enrolled to the study. Of the patients recruited; 16 (44.4%) had gliomas (astrocytoma, oligodendroglioma, and ependymoma), 12 (33.3%) had primitive neuroendrocrine tumours/medulloblastomas, 5 (13.9%) had pineal tumours (pineal germinomas), 2 (5.6%) had chondrosarcomas, and 1 (2.8%) had a brain stem tumour with no formal histological diagnosis. Diagnosis and treatment of the tumour was during childhood in 16 and AO in 20. The mean radiotherapy dose to the tumour was 51.9 ± 10.1 Gy. Endocrine deficits were highly prevalent within the cohort (severe growth hormone deficiency (GHD) *n* = 22; partial GHD (*n* = 10); adrenocorticotrophic hormone deficiency (*n* = 1); hypogonadism (n = 7); and hypothyroidism (n = 7)). All patients with deficiencies were on appropriate replacement therapy, with the exception of GHD, where 10/22 were receiving GH replacement therapy.

### Overall cohort

Patients demonstrated abnormal body composition characterized by an increase in BMI (27.2 ± 6.4 vs 24.1 ± 3.3 kg/m^2^, *P* = 0.01), waist circumference (93.9 ± 15.6 vs 80.3 ± 10.9 cm, *P* < 0.001), and bioimpedance measures of fat mass compared with controls. Total body fat mass (bioimpedance) was increased in the patient cohort (24.0 ± 12.2 vs 15.7 ± 6.6 kg, *P* < 0.001), as was central fat mass (increased waist circumference and bioimpedance truncal fat mass, 13.0 ± 6.7 vs 8.2 ± 3.7 kg, *P* < 0.001). The increase in bioimpedance truncal fat mass per cent was of similar magnitude to the overall increase in total fat mass per cent. Significant increases in skinfold measurements of subcutaneous fat mass were observed at all sites measured. Results are presented in Table 1.

**Table 1:** Body composition of brain tumour survivors and age- and gender-matched controls.

	Patients	Controls	*P*
BMI	27.2 ± 6.4	24.1 ± 3.3	**0.010**
Waist (cm)	93.9 ± 15.6	80.3 ± 10.9	**<0.001**
WHR	0.88 ± 0.08	0.82 ± 0.08	**0.001**
BIA FM (kg)	24.0 ± 12.2	15.7 ± 6.6	**<0.001**
BIA FM%	29.6 ± 9.7	22.1 ± 8.3	**<0.001**
BIA LBM (kg)	54.1 ± 13.5	54.0 ± 12.0	0.96
BIA truncal FM (kg)	13.0 ± 6.7	8.2 ± 3.7	**<0.001**
BIA truncal FM%	29.4 ± 10.0	21.0 ± 8.1	**<0.001**
BIA truncal LBM (kg)	29.3 ± 6.7	31.4 ± 9.2	0.29
SKF suprailiac (mm)	21.1 ± 10.7	10.6 ± 5.1	**<0.001**
SKF infrascapular (mm)	22.5 ± 9.9	13.9 ± 6.2	**<0.001**
SKF biceps (mm)	13.6 ± 5.8	8.4 ± 4.2	**<0.001**
SKF triceps (mm)	18.4 ± 9.2	10.1 ± 5.2	**<0.001**
Sum of SKF (mm)	75.7 ± 32.3	42.5 ± 16.4	**<0.001**

BIA, bioimpedance; BMI, body mass index; FM, fat mass; LBM, lean body mass; SKF, skinfold; WHR, waist–hip ratio.

The patient cohort showed a significantly greater burden of vascular risk factors compared with the control group. Patients had an adverse lipid profile as evident by higher total cholesterol (5.3 ± 1.1 vs 4.6 ± 1.0 mmol/L, *P* = 0.007) and LDL cholesterol levels (3.1 ± 0.8 vs 2.7 ± 0.9 mmol/L, *P* < 0.011). Although fasting glucose levels were similar in the two groups, patients had increased fasting insulin levels (13.4 ± 13.1 vs 7.6 ± 3.3 miu/L, *P* = 0.014), suggesting the presence of insulin resistance. This was supported further by calculating the HOMA-IR index, which was significantly elevated in the patient cohort (2.9 ± 2.84 vs 1.66 ± 0.73, *P* = 0.016). No difference in the 24-h ambulatory BP, either systolic or diastolic, was observed. Further analysis of ambulatory BP after stratification into daytime and nocturnal BP showed no significant differences between patients and control subjects. Table 2 summarizes the above laboratory results.

**Table 2:** Metabolic parameters of brain tumour survivors and age- and gender-matched controls.

	Patients	Controls	*P* value
Total cholesterol (mmol/L)	5.3 ± 1.1	4.6 ± 1.0	**0.007**
LDL-cholesterol (mmol/L)	3.1 ± 0.8	2.7 ± 0.9	**0.011**
HDL-cholesterol (mmol/L)	1.6 ± 0.6	1.4 ± 0.4	0.14
Triglycerides (mmol/L)	1.7 ± 2.7	1.1 ± 0.5	0.17
Fasting glucose (mmol/L)	4.8 ± 0.8	4.8 ± 0.4	0.60
Fasting insulin (miu/L)	13.4 ± 13.1	7.6 ± 3.3	**0.014**
HOMA-IR	2.90 ± 2.84	1.66 ± 0.73	**0.016**
24-h Systolic BP (mmHg)	117 ± 13.0	117 ± 9.3	0.99
24-h Diastolic BP (mmHg)	65.5 ± 9.0	64.4 ± 6.2	0.58

BP, blood pressure; LDL, low-density lipoprotein; HDL, high-density lipoprotein; HOMA-IR, homeostatic model assessment for insulin resistance.

**Table 3:** Body composition of adult and childhood brain tumour survivors and age- and gender-matched controls.

	AO patients	AO controls	*P*	CO patients	CO controls	*P*
BMI (kg/m^2^)	28.0 ± 6.0	25.4 ± 3.4	0.107	26.1 ± 6.9	22.5 ± 2.5	0.309
Waist (cm)	96.0 ± 16.0	85.1 ± 11.5	**0.018**	91.2 ± 15.3	74.3 ± 6.5	**<0.001**
WHR	0.89 ± 0.09	0.84 ± 0.08	0.092	0.88 ± 0.07	0.79 ± 0.09	**<0.001**
BIA FM (kg)	25.2 ± 12.1	18.3 ± 7.0	0.060	22.5 ± 12.4	12.5 ± 4.5	**0.012**
BIA FM%	30.0 ± 10.6	24.3 ± 8.3	0.065	29.0 ± 8.8	19.3 ± 7.6	**0.007**
BIA LBM (kg)	56.3 ± 12.2	55.9 ± 12.5	0.928	51.4 ± 15.0	51.6 ± 11.2	0.665
BIA truncal FM (kg)	14.1 ± 6.9	10.0 ± 3.8	**0.029**	11.6 ± 6.3	6.3 ± 2.6	**0.005**
BIA truncal FM%	30.6 ± 10.8	24.0 ± 6.8	**0.031**	27.9 ± 9.1	17.5 ± 8.4	**0.003**
BIA truncal LBM (kg)	30.3 ± 6.2	30.9 ± 5.4	0.76	28.0 ± 7.3	32.0 ± 12.4	0.30
SKF suprailiac (mm)	22.8 ± 12.1	12.2 ± 5.1	**<0.001**	19.2 ± 9.0	8.5 ± 4.5	**<0.001**
SKF infrascapular (mm)	22.3 ± 10.9	16.2 ± 6.7	**0.050**	22.8 ± 9.0	11.0 ± 4.1	**<0.001**
SKF biceps (mm)	14.6 ± 6.2	9.0 ± 4.1	**0.002**	12.5 ± 5.3	7.6 ± 4.4	**0.007**
SKF triceps (mm)	19.1 ± 10.1	11.2 ± 5.2	**0.004**	17.6 ± 8.3	8.8 ± 5.1	**0.001**
Sum of SKF (mm)	78.8 ± 36.6	47.8 ± 15.4	**0.001**	72.2 ± 27.5	35.8 ± 15.7	**<0.001**

BIA, bioimpedance; BMI, body mass index; FM, fat mass; LBM, lean body mass; SKF, skinfold; WHR, waist–hip ratio.

**Table 4:** Metabolic parameters of adult and childhood brain tumour survivors and age- and gender-matched controls.

	AO patients	AO controls	*P*	CO patients	CO controls	*P*
Total cholesterol (mmol/L)	5.6 ± 1.2	4.9 ± 1.0	**0.036**	5.0 ± 0.9	4.3 ± 0.9	0.054
LDL-cholesterol (mmol/L)	3.2 ± 0.8	2.9 ± 1.0	0.185	3.0 ± 0.8	2.4 ± 0.7	**0.044**
HDL-cholesterol (mmol/L)	1.8 ± 0.7	1.4 ± 0.4	0.133	1.4 ± 0.4	1.5 ± 0.4	0.906
Triglycerides (mmol/L)	1.4 ± 0.6	1.2 ± 0.6	0.117	2.1 ± 3.9	1.0 ± 0.4	0.579
Fasting glucose (mmol/L)	5.1 ± 1.0	4.9 ± 0.4	0.828	4.6 ± 0.4	4.6 ± 0.4	0.642
Fasting insulin (miu/L)	15.6 ± 16.6	8.5 ± 3.3	0.069	10.6 ± 5.8	6.5 ± 2.8	**0.019**
HOMA (IR)	3.53 ± 3.61	1.84 ± 0.75	**0.047**	2.11 ± 1.08	1.40 ± 0.64	**0.038**
24-h Systolic BP (mmHg)	119 ± 13.4	119 ± 9.1	0.90	115 ± 12.9	115 ± 9.5	0.99
24-h Diastolic BP (mmHg)	66.5 ± 8.6	65.8 ± 5.9	0.79	64.6 ± 9.6	62.7 ± 6.3	0.53

BP, blood pressure; HDL, high-density lipoprotein; HOMA-IR, homeostatic model assessment for insulin resistance; LDL, low-density lipoprotein.

### Effect of timing of malignant tumour diagnosis

Patients were stratified into those of CO (*n* = 16, mean age 20.4 ± 3.8 years at the time of enrolment to the study, 7 female) and AO (*n* = 20, age 39.3 ± 13.3 years, 8 female) brain tumour survivors and were compared with their respective matched controls (CO controls, *n* = 16, age 21.8 ± 3.8 years, 7 female; AO controls, *n* = 20, age 39.4 ± 13.1, 8 female) (Tables 3 and 4).

AO patients demonstrated a less favourable body composition, which was characterized by an increase in central fat mass (waist, BIA truncal FM and truncal FM%); however, total body fat mass measured by BMI and BIA FM and FM% were not different to controls. Subcutaneous fat (expressed as skinfold thickness) of the AO patients was increased at all sites. Both systolic and diastolic BPs were similar in AO patients and controls. Compared with their controls, AO patients had significantly higher total cholesterol levels (5.6 ± 1.2 vs 4.9 ± 1.0 mmol/L, *P* = 0.036), but no difference was found in LDL-C, HDL-C or triglyceride levels. One AO patient had a diagnosis of type 2 diabetes. Fasting glucose and insulin levels were not elevated; however, insulin resistance as measured by HOMA was increased (3.53 ± 3.61 vs 1.84 ± 0.75, *P* = 0.047).

Total body fat mass (BIA FM and FM%), truncal fat mass (waist, WHR, BIA truncal FM and FM%), and subcutaneous fat mass (regional and total SKFs) were significantly elevated in CO patients compared with controls. No difference in systolic or diastolic BP was observed between CO patients and controls. The lipid profile of CO patients showed significantly increased LDL-C compared with their controls (3.0 ± 0.8 vs 2.4 ± 0.7 mmol/L, *P* = 0.044), but no difference in total, HDL-C, or triglyceride levels. One CO patient had a diagnosis of type 2 diabetes. Fasting glucose levels were not significantly different, whereas fasting insulin levels and insulin resistance were increased in the CO patients (10.6 ± 5.8 vs 6.5 ± 2.8 miu/L, *P* = 0.019 and 2.11 ± 1.08 vs 1.4 ± 0.64, *P* = 0.038, respectively). Combining the measured variables, two patients in each of the AO patients, AO controls, CO patients, and none in the CO controls were defined as having metabolic syndrome.

For each endpoint, the mean differences between the AO patients and their controls were calculated. This was similarly undertaken for the CO patients and controls. Comparison of the mean differences of the AO and CO cohorts was non-significant for all study variables.

### Correlations

Predictors of the excess vascular risk markers within the brain tumour cohort were examined using univariate correlations and multivariate regression analyses. Multivariate models contained the independent variables of age, gender, timing of onset (AO or CO), tumour irradiation dosage, and time from diagnosis. Additionally, as endocrine gland dysfunction was prevalent within the cohort and is recognized to influence vascular risk, the presence or absence of deficiency was added to the model. Not all GHD patients were on GH replacement and GH replacement, or the absence of, was therefore added to the model.

Dependent variables examined were total cholesterol, LDL-C, total body fat mass, truncal fat mass, sum of skinfolds, and HOMA-IR index. No significant univariate correlations were detected, and within multivariate analyses there were no significant predictors of the dependent variables.

## Discussion

In this pilot study, we show survivors of primary malignant brain tumours appear to have an increased prevalence of adverse vascular risk factors. Specifically, these patients exhibited changes in body composition defined by a greater proportion of body fat which was both central and subcutaneous; increased total and LDL-cholesterol; and insulin resistance. No concomitant increase in BP was observed. The excess of adverse vascular risk factors was observed in the overall cohort, as well as in the sub-cohorts of survivors of malignant brain tumours of both AO and CO. The degree of abnormality in vascular risk factors was similar after the division of the cohort into those of CO and AO. To our knowledge, vascular risk factors have not previously been studied in survivors of adult malignant brain tumours.

Previous studies have been derived primarily from childhood cancer survivors (CCS) of mixed aetiology or those with acute lymphoblastic leukaemia (ALL) and have consistently shown increased prevalence of vascular risk factors. Abnormalities of body composition in CCS are well documented and characterized by an increased risk of obesity (11, 12, 13, 14). Talvensaari and colleagues compared 50 CCS with age- and sex-matched controls and showed an increased prevalence of obesity (16 vs 5%) and body FM (25% vs. 20%) (15). Similarly, assessment of FM in survivors of childhood ALL showed increased total body FM, with more than one in four patients having an FM greater than the 90th centile of the reference values (16). The prevalence of truncal adiposity, which has the strongest association with components of the metabolic syndrome, is estimated to be around two-fold greater in CCS than that of healthy controls (17). There are few data in CCS regarding ambient lipid profiles. Talvesaari and colleagues described reduced HDL-C and total cholesterol levels, with a resultant increase in the TC:HDL ratio in CCS. LDL-C and triglyceride levels of the CCS were however normal (15). In 23 recipients of childhood bone marrow transplant (BMT) for haematological malignancies (*n* = 17) or aplastic anaemia/myelodysplasia, 9 had hypertriglyceridaemia and 5 had low HDL values. Only mean triglyceride values were, however, significantly different from the control group (18).

In a mixed cohort of CCS where BPs were compared with standards for age, gender, and height, 28% of CCS were observed to have a systolic or diastolic BP above the 90th centile (14). The highest prevalence of hypertension, unsurprisingly, occurred in patients with a primary diagnosis of Wilm’s tumour (14). A further study reported a four-fold increase in the frequency of hypertension in a mixed cohort of CCS (17), however, a number of studies have failed to detect any abnormality of BP (15). Insulin resistance is fundamental to metabolic syndrome and conveys a significantly elevated risk of vascular disease. Fasting glucose and insulin are reported to be elevated in both long-term CCS (15) and recipients of BMT during childhood (18). In a prospective study of glucose tolerance in 248 CCS, the recorded frequencies of hyperinsulinaemia, impaired glucose tolerance, and diabetes were significantly elevated at 18% (17). Where insulin resistance has been described in CCS, an association with obesity, subnormal HDL levels, or hypertriglyceridaemia is frequent (17, 18), raising the question as to whether these individuals have metabolic syndrome.

These data may, however, not necessarily be transferrable to patients treated for childhood malignant brain tumours (SCBT) given the differences in treatment regimens. In comparison with other common childhood malignancies (leukaemia, lymphoma, Wilm’s tumour, neuroblastoma, soft tissue sarcoma), children with malignant brain tumours more frequently receive surgery and cranial radiation, and traditionally chemotherapy has been used sparingly. It is important, therefore, to establish the impact of the different disease processes and treatment regimens used for malignant brain tumours on the prevalence of vascular risk factors.

Data specific to long-term SCBT has variably shown increases in the prevalence of obesity (7) or no difference to reference populations (19). When direct measures of fat mass are used, CBTS have increased total body FM%, truncal FM, and appendicular FM (7), and reduced LBM (7). A recent systematic review and meta-analysis of 17 studies including a total of 2032 SCBT failed to show an increase in the prevalence of overweight or obesity compared with non-cancer controls (20). The authors, however, went on to analyse six studies with data on adiposity and showed SCBT to have increased WHR and per cent FM. Our data are in keeping with the data on SCBT showing increases in adiposity but extend this further to show increases in both truncal and subcutaneous FM. Truncal FM was increased by just over 80% in the childhood survivors and 40% in AO survivors, the difference likely relating to higher truncal fat mass in the adult controls.

In the few studies of SCBT that have examined serum lipids, an increase in LDL-C, apolipoprotein-B, total cholesterol to HDL ratio, triglycerides, and reduction in HDL-C has been reported (6, 7). No differences in lipid levels are observed between those with exposure of the hypothalamus to external beam radiation (XRT), or not (7). Insulin resistance estimated by the HOMA-IR has been found to be higher in SCBT who received hypothalamic XRT, compared with those who were not exposed (7). Our cohort showed increases in total and LDL-C but without the accompanying changes in HDL-C or triglycerides previously reported. The latter is difficult to explain given the significant increase in truncal fat mass and insulin resistance in our cohort. Notably, similar adverse changes in lipid profile and insulin sensitivity were observed in both our CO and AO survivors. Survivors of childhood brain cancer are reported to have isolated systolic hypertension in one study (6). No difference in BP is present in those exposed and not exposed to hypothalamic XRT (7). Using 24-h ambulatory BP monitoring, we could not demonstrate a difference between patients and control subjects for our overall cohort, or after stratification by the timing of onset of the brain tumour.

The observation of increased vascular risk factors in CBTS is given importance by data showing CBTS to have a standardized mortality rate (SMR) of 4.2 for cardiac deaths (3), and increased risk of late-occurring stroke (RR 29.0) (5). Similar findings have been reported in unselected cohorts of long-term CCS for both cardiac (SMR 5.8–8.2) (21, 22, 23) and cerebrovascular disease (SMR 4.6) (22). Data in teenage and young adult cancer survivors (age 15–39 years) have shown a 30 and 40% increased rate of admission to hospital with cardiovascular disease (9) and cerebrovascular events (8) respectively, confirming excess vascular disease continues to be a significant late sequelae in cancer survivors with increasing age at diagnosis. We are unaware of similar data in purely adult brain tumour survivors and suspect this relates to the relatively poor prognosis of these individuals with age at diagnosis. With further improvements in the duration of survival of adult-onset brain tumours, however, cardiovascular and cerebrovascular diseases are likely to take on increasing relevance.

Hypothalamic damage resulting from the tumour, surgery, or radiotherapy has been implicated as the primary risk factor in the development of obesity in CBTS (24). In keeping with this finding the authors showed hypothalamic tumour location, hypothalamic irradiation doses > 51 Gy and endocrinopathies (particularly GHD) to be associated with the development of obesity in CBTS (24). Younger age at diagnosis also increased the risk of obesity, suggesting the younger brain to be more susceptible to hypothalamic damage (19, 24). Comparison of CBTS exposed to hypothalamic XRT (>1 Gy) to those not exposed to XRT show similar BMI, total body FM, and %FM, however those with exposure of the hypothalamus to XRT have greater visceral, but not subcutaneous, fat, and metabolic sequelae (7). It has been postulated that the greater visceral fat and GHD may at least in part explain the increased metabolic sequelae (7).

Examining previous data concerning vascular risk factors in brain tumour survivors (6, 7, 19, 20) and accepting that all previous data have been in SCBT, our cohort appears to show a greater degree of difference compared with the reference population. Possible explanations for this observation are that our entire cohort received high dose cranial XRT and almost all patients showed hormone deficits, in particular GHD (25). GHD individuals show increases in truncal fat mass, total and LDL-C, and insulin resistance (26, 27). Whether GH replacement can reverse the observed metabolic abnormalities in survivors of brain tumours is unclear. Our previous work showed GH replacement in adult survivors of childhood cancer of mixed aetiology led to minor improvements in body composition and serum lipids (28), suggesting GHD likely contributes to the overall adverse cardiovascular risk profile but is unlikely to be the only determinant. The primary limitation of our study relates to the small numbers within our cohort, however, provides a platform to support further examination of cardiovascular risk factors in a larger cohort of brain tumour survivors. Additionally, our cohort is heterogeneous in terms of subtypes of brain tumours and treatment regimens, though given the low incidence of many childhood brain tumours and continued evolution of multi-modality treatment regimens for all brain tumours, it is unlikely a sufficiently large study of long-term survivors with a single tumour pathology and single treatment regimen will be possible. A further confounder is that our control group may not be fully representative of the background population. The mean BMI of our control group was 24.1 kg/m^2^ which is lower than that reported in larger population studies in the UK (29) and therefore may have amplified differences between our patients and controls. Our data are cross-sectional and would be strengthened by longitudinal data to determine timelines for change in vascular risk factors in our patients. Such a study would, however, require recruitment of patients at the time of definitive treatment of their tumour and prolonged follow-up.

In summary, in this pilot study, we have provided data that suggest survivors of malignant brain tumours have an adverse cardiovascular risk profile characterized by a combination of excess total body and truncal fat mass, elevated total and LDL-cholesterol, and insulin resistance. We found these anomalies to be present in brain tumour survivors of both CO and AO. These observations are in keeping with studies showing increases in cardiovascular and cerebrovascular disease in cancer survivors diagnosed in childhood, adolescent, and young adult life. To our knowledge, these data are the first to examine the cardiovascular risk profile of AO brain tumour survivors. As survival rates improve in AO brain tumours our findings are likely to derive increasing importance and therefore warrant further study.

## Declaration of interest

The authors declare that there is no conflict of interest that could be perceived as prejudicing the impartiality of the research reported.

## Funding

This work did not receive any specific grant from any funding agency in the public, commercial, or not-for-profit sector.

## Author contribution statement

SSK, RA, JL, & RDM were involved with study design and conceptualization. NK, MG, KS, JL, and RDM were involved with data curation and formal analysis. NK, SSK, KS, GKD, HR, AG, MK-W, GG, CL, AA, JL, and RDM undertook investigation and methodology (including patient recruitment, screening, consent, laboratory analyses, patient investigations). SSK, NK, KS and JL undertook day to day project administration. RA and RDM undertook overall supervision of the laboratory analysis and research team. NK produced the - original draft, which was edited and reviewed by RA and RDM before being sent to the other investigators for review. All investigators were involved with review and editing of the draft to achieve the final manuscript.
